# Supplementary material for: Evaluating the impact of COVID-19 pandemic on complicated malaria admissions and outcomes in the paediatric Ho Teaching Hospital of the Volta Region of Ghana
Source: PLOS Glob Public Health. 2022 Sep 15;2(9):e0000509. doi: 10.1371/journal.pgph.0000509 (PMC10021415; doi:10.1371/journal.pgph.0000509)
Supplement: Attachment — Submitted filename: Detailed response to reviewer PLOS GBH.docx [file pgph.0000509.s001.docx]

**Response to Editor and Reviewers**

**We the authors are very grateful to the Editor and reviewers for these important comments and suggestions to enrich our paper.**

**Below is our response to the comments.**

1. You indicated that you had ethical approval for your study. In your Methods section, please ensure you have also stated whether you obtained consent from parents or guardians of the minors included in the study or whether the research ethics committee or IRB specifically waived the need for their consent.

**Response: this is a retrospective study consent form was not applicable.**

2. Please amend your Financial Disclosure statement. If you did not receive any funding for this study, please simply state: “The authors received no specific funding for this work.”

**Response: This has been done. Thank you**

3. Please update your Competing Interests statement. If you have no competing interests to declare, please state: “The authors have declared that no competing interests exist.”

**Response: This has been done. Thank you**

4. In the online submission form, you indicated that “DATA ARE AVAILABLE FROM THE HOSPITAL RECORD AND WILL BE PROVIDED ON REQUEST”. All PLOS journals now require all data underlying the findings described in their manuscript to be freely available to other researchers, either 1. In a public repository, 2. Within the manuscript itself, or 3. Uploaded as supplementary information.
This policy applies to all data except where public deposition would breach compliance with the protocol approved by your research ethics board. If your data cannot be made publicly available for ethical or legal reasons (e.g., public availability would compromise patient privacy), please explain your reasons by return email and your exemption request will be escalated to the editor for approval. Your exemption request will be handled independently and will not hold up the peer review process, but will need to be resolved should your manuscript be accepted for publication. One of the Editorial team will then be in touch if there are any issues.

**Response: This has been done. Thank you. Letter explaining reasons for our inability to provide our data source will be sent to the editor.**

5. Please provide separate figure files in .tif or .eps format only and remove any figures embedded in your manuscript file. Please ensure that all files are under our size limit of 20MB.

For more information about how to convert your figure files please see our guidelines: <https://journals.plos.org/globalpublichealth/s/figures>

**Response: This has been done**

Additional Editor Comments (if provided):

1. Reference are not codded properly. E.g. see reference-19 coated in the text page no-4.

**Response: this has been corrected. Thank you**

2. Found capital letters in Abruptly in the document. e.g. page-3 Severe malaria in 2nd paragraph; page-4 Outpatient, Department.

**Response: this has been corrected. Thank you**

3. Update the disease burden with latest reports.

**Response: this has been done. Thank you**


[Note: HTML markup is below. Please do not edit.]

Reviewers' comments:

Reviewer's Responses to Questions

**Comments to the Author**

1. Does this manuscript meet PLOS Global Public Health’s [publication criteria](https://journals.plos.org/globalpublichealth/s/criteria-for-publication)? Is the manuscript technically sound, and do the data support the conclusions? The manuscript must describe methodologically and ethically rigorous research with conclusions that are appropriately drawn based on the data presented.

Reviewer #1: Yes

Reviewer #2: Partly

2. Has the statistical analysis been performed appropriately and rigorously?

Reviewer #1: Yes

Reviewer #2: No

3. Have the authors made all data underlying the findings in their manuscript fully available (please refer to the Data Availability Statement at the start of the manuscript PDF file)?

The [PLOS Data policy](https://journals.plos.org/globalpublichealth/s/data-availability) requires authors to make all data underlying the findings described in their manuscript fully available without restriction, with rare exception. The data should be provided as part of the manuscript or its supporting information, or deposited to a public repository. For example, in addition to summary statistics, the data points behind means, medians and variance measures should be available. If there are restrictions on publicly sharing data—e.g. participant privacy or use of data from a third party—those must be specified.

Reviewer #1: Yes

Reviewer #2: Yes

4. Is the manuscript presented in an intelligible fashion and written in standard English?

PLOS Global Public Health does not copyedit accepted manuscripts, so the language in submitted articles must be clear, correct, and unambiguous. Any typographical or grammatical errors should be corrected at revision, so please note any specific errors here.

Reviewer #1: Yes

Reviewer #2: Yes

5. Review Comments to the Author

Please use the space provided to explain your answers to the questions above. You may also include additional comments for the author, including concerns about dual publication, research ethics, or publication ethics. (Please upload your review as an attachment if it exceeds 20,000 characters)

Reviewer #1: The study provides valuable information on the impact of the COVID19 pandemic among the children affected with complicated malaria.

**Response: Thank you very much**

However, the following points should be addressed to make it more informative and understanding.
1. Has covid 19 impacted the approach of patients to admit to the hospital?

**Response: this paper is highlighting the possibility of the COVID-19 pandemic decreasing health seeking behaviour among parents of children suffering from complicated malaria**

2. Page no. 6: “Malaria caused over 270 thousand childhood deaths in 2018, constituting about 67% of all malaria deaths globally” [11]:-In the interest of readers, WHO Malaria report 2021 should also be cited.

**Response: thank you so much this has been added.**

3. Page no. 7 “With malaria among children still a significant public health issue in Ghana19, no study has looked at the impact of the pandemic on this.” The sentence should be revised.

**Response: this section has been revised. Thank you very much**

4. Page no. 11: In table 1, CM and SMA should be described as Cerebral malaria (CM) and severe malaria anemia (SMA).

**Response: this has been done. Thank you very much**

Optionally, the information on the status of malaria in the country and national treatment guidelines for the treatment of children should also be addressed.

**Response: thank for the suggestion however we the authors of the paper prefers not to proceed with this option**

Reviewer #2: The manuscript is interesting and well written.

**Response: thank you very much**

However it is unclear how the pandemic may have changed the malaria burden among children. Perhaps access to healthcare, but unlikely the true burden (prevalence). **Response: the focus of this manuscript is on the possible impact of COVID-19 pandemic on the health seeking behavior and access to healthcare among children with complicated malaria and not on the prevalence or burden of complicated among children in the population.**

The authors should clarify the true objectives and distinguish between what they are measuring and the underlying mechanism.

**Response: this has been clearly stated in the manuscript. Thank you.**

I have several comments that should be addressed:

Typo on the last sentence on page 3 - the sentence ends with ‘the COVID-19’ where it should read ‘the COVID-19 pandemic’.

**Response: this has been corrected. Thank you very much**

In the intro page 3, it is unclear why the authors cite hospital visits from prior to control programs, when the baseline of hospital visits for this study will come from the era of malaria control programs. These are now outdated statistics

**Response: authors believe that these are historical statistical representations that importantly highlight the trend of malaria burden.**

Specie should be species.

**Response: thank you. Change has been effected**

Cerebral and Severe do not need to be capitalized

**Response: this has been corrected**. Thank you

The COVID pandemic may have created an additional barrier to healthcare seeking, but it certainly is not the only barrier and did not create these barriers.

**Response: this has been inputted into the manuscript. Thank you.**

Last paragraph page 3 - first 2 cases of COVID, it just says first 2 cases without specifying cases of what.

**Response: This has been inputted. Thank you**

The authors should define what measures were included in the lock down, this is a fairly generic term.

**Response: this has been added into the manuscript. Thank you**

For the yearly differences, using a Pearson Chi2 test will test for differences between all of the years. Therefore the results don’t match with the analysis. For example, the authors state that there were significantly more children admitted with cerebral malaria in 2020 compared to other year. The chi2 test would not show this, just that there were differences between the years, not that 2020 was statistically significantly higher than all the others. This would be better shown and interpretable by having confidence intervals in table 2 to see if 2020 was different from the other years. As it is, all the table tells is that the years are different from each other.

The graphs should also have confidence bands or intervals to show the statistical significance, rather than just p values.

**Response: this has been done. Thank very much**

6. PLOS authors have the option to publish the peer review history of their article ([what does this mean?](https://journals.plos.org/globalpublichealth/s/editorial-and-peer-review-process#loc-peer-review-history)). If published, this will include your full peer review and any attached files.


**Do you want your identity to be public for this peer review?** If you choose “no”, your identity will remain anonymous but your review may still be made public.


For information about this choice, including consent withdrawal, please see our [Privacy Policy](https://www.plos.org/privacy-policy).

Reviewer #1: **Yes:** Kuldeep Singh

Reviewer #2: No
