# Supplementary material for: Evaluating the impact of COVID-19 pandemic on complicated malaria admissions and outcomes in the paediatric Ho Teaching Hospital of the Volta Region of Ghana
Source: PLOS Glob Public Health. 2022 Sep 15;2(9):e0000509. doi: 10.1371/journal.pgph.0000509 (PMC10021415; doi:10.1371/journal.pgph.0000509)
Supplement: Attachment — Submitted filename: RESPONSE TO EDITOR AND REVIEWER.docx [file pgph.0000509.s002.docx]

**RESPONSE TO EDITOR AND REVIEWER**

**We the authors are very grateful to the Editor and reviewer for these important comments and suggestions to enrich our paper.**

**Below is our response to the comments.**

Journal Requirements:

1. Please review your reference list to ensure that it is complete and correct. If you have cited papers that have been retracted, please include the rationale for doing so in the manuscript text, or remove these references and replace them with relevant current references. Any changes to the reference list should be mentioned in the rebuttal letter that accompanies your revised manuscript. If you need to cite a retracted article, indicate the article’s retracted status in the References list and also include a citation and full reference for the retraction notice.

**RESPOSNSE: To the best of our knowledge we the authors believe that references are correct and complete.**

Additional Editor Comments (if provided):

[Note: HTML markup is below. Please do not edit.]

Reviewers' comments:

Reviewer's Responses to Questions

**Comments to the Author**

1. If the authors have adequately addressed your comments raised in a previous round of review and you feel that this manuscript is now acceptable for publication, you may indicate that here to bypass the “Comments to the Author” section, enter your conflict of interest statement in the “Confidential to Editor” section, and submit your "Accept" recommendation.

Reviewer #1: All comments have been addressed

Reviewer #2: (No Response)

2. Does this manuscript meet PLOS Global Public Health’s [publication criteria](https://journals.plos.org/globalpublichealth/s/criteria-for-publication)? Is the manuscript technically sound, and do the data support the conclusions? The manuscript must describe methodologically and ethically rigorous research with conclusions that are appropriately drawn based on the data presented.

Reviewer #1: Yes

Reviewer #2: Yes

3. Has the statistical analysis been performed appropriately and rigorously?

Reviewer #1: Yes

Reviewer #2: Yes

4. Have the authors made all data underlying the findings in their manuscript fully available (please refer to the Data Availability Statement at the start of the manuscript PDF file)?

The [PLOS Data policy](https://journals.plos.org/globalpublichealth/s/data-availability) requires authors to make all data underlying the findings described in their manuscript fully available without restriction, with rare exception. The data should be provided as part of the manuscript or its supporting information, or deposited to a public repository. For example, in addition to summary statistics, the data points behind means, medians and variance measures should be available. If there are restrictions on publicly sharing data—e.g. participant privacy or use of data from a third party—those must be specified.

Reviewer #1: Yes

Reviewer #2: Yes

5. Is the manuscript presented in an intelligible fashion and written in standard English?

PLOS Global Public Health does not copyedit accepted manuscripts, so the language in submitted articles must be clear, correct, and unambiguous. Any typographical or grammatical errors should be corrected at revision, so please note any specific errors here.

Reviewer #1: Yes

Reviewer #2: Yes

6. Review Comments to the Author

Please use the space provided to explain your answers to the questions above. You may also include additional comments for the author, including concerns about dual publication, research ethics, or publication ethics. (Please upload your review as an attachment if it exceeds 20,000 characters)

Reviewer #1: The authors have addressed all the issues and seems interesting to the scientific community.

**RESPONSE: thank you very much**

Reviewer #2: Most of the comments from the previous review have been addressed.

**RESPONSE: Thank you very much**

However there are still some minor revisions that should be addressed. There are some typos and errors in language that should be corrected before publication. Those are:

Abstract: Page 2 - ‘Children admitted in 2020 had the shorted mean stay on admission’. Should be corrected to shortest.

**RESPONSE: this has been done**

Severe Malaria and Cerebral Malaria are still capitalized throughout when they shouldn’t be.

**RESPONSE**: **this has been done**

Should read ‘The majority of children’ instead of ‘Majority of children’

**RESPONSE**: **this has been done**

There is an extra parentheses in the third paragraph of the discussion.

**RESPONSE**: **this has been removed**

In the fourth paragraph of the discussion it should read ‘The majority of children’ rather than ‘majority of children’.
**RESPONSE**: **this has been done**

Additionally:

Introduction: first paragraph. When citing the COVID statistics of cases and deaths it would be helpful to reference the time period, such as , ‘as of June 2022’ since these are going up every day.

**RESPONSE**: **this has been done. Thank you.**

Suggest editing the last sentence of page 4 to ‘might have increased these barriers’. The pandemic didn’t create all the barriers to healthcare and health seeking as the authors describe earlier in the paragraph. Many barriers exist, but the pandemic didn’t create all of these barriers, but added substantially to them.

**RESPONSE**: **some revision have been done. Thank you. We the authors agree with you that the pandemic did not create all the barriers but might have caused additional barriers or worsened already existing barriers as stated in the manuscript. Thank you very much.**

7. PLOS authors have the option to publish the peer review history of their article ([what does this mean?](https://journals.plos.org/globalpublichealth/s/editorial-and-peer-review-process#loc-peer-review-history)). If published, this will include your full peer review and any attached files.


**Do you want your identity to be public for this peer review?** If you choose “no”, your identity will remain anonymous but your review may still be made public.


For information about this choice, including consent withdrawal, please see our [Privacy Policy](https://www.plos.org/privacy-policy).

Reviewer #1: No

Reviewer #2: No

[NOTE: If reviewer comments were submitted as an attachment file, they will be attached to this email and accessible via the submission site. Please log into your account, locate the manuscript record, and check for the action link "View Attachments". If this link does not appear, there are no attachment files.]

While revising your submission, please upload your figure files to the Preflight Analysis and Conversion Engine (PACE) digital diagnostic tool, https://pacev2.apexcovantage.com/. PACE helps ensure that figures meet PLOS requirements. To use PACE, you must first register as a user. Registration is free. Then, login and navigate to the UPLOAD tab, where you will find detailed instructions on how to use the tool. If you encounter any issues or have any questions when using PACE, please email PLOS at figures@plos.org. Please note that Supporting Information files do not need this step.
